# Impact of the number of repeated inhalations and patient characteristics on the residual amount of inhaled laninamivir octanoate hydrate dry powder in pediatric patients with influenza

**DOI:** 10.1186/s40780-017-0094-7

**Published:** 2017-11-08

**Authors:** Toshiki Murasaka, Kenji Ikemura, Tomoyuki Enokiya, Yuichi Muraki, Mayumi Ikemura, Koji Terada, Takuya Iwamoto, Masahiro Okuda

**Affiliations:** 1 Konan Pharmacy, Tsu, Mie 514-0323 Japan; 2 Department of Clinical Pharmacy and Biopharmaceutics, Mie University Graduate School of Medicine, Tsu, Mie 514-8507 Japan; 3 Department of Pharmacy, Mie University Hospital, 2-174, Edobashi, Tsu, Mie 514-8507 Japan; 4 Tsu Pharmaceutical Association, Tsu, Mie 514-1135 Japan

**Keywords:** Inhalation, Influenza, Laninamivir, Peak inspiratory flow, Pediatric patients

## Abstract

**Background:**

A dry powder inhaled formulation is used for the anti-influenza drug laninamivir octanoate hydrate (laninamivir). Although two successive inhalations (puffs) are recommended to minimize residual amounts of active ingredients, previous reports suggest that pediatric patients with low peak inspiratory flow are unable to inhale the active ingredient adequately. In the present study, we prospectively investigated the appropriate number of repeated inhalations of laninamivir dry powder and factors influencing the residual amount of ingredients in pediatric patients with influenza.

**Methods:**

The study enrolled 64 patients receiving laninamivir dry powder inhaler (Inavir®) between January and March 2016 at Tsu emergency medical center/pediatric clinic and dental clinic. All patients enrolled used a laninamivir dry powder inhaler in four repeated inhalations, as instructed by a pharmacist. The residual amount of laninamivir dry powder was calculated by measuring the device weight before and after each inhalation and a residual amount of >20% was defined as an unsuccessful inhalation.

**Results:**

The inadequate inhalation rate after two successive inhalations was 45%, and it decreased as number of inhalation repeats increased, reaching 23% after four successive inhalations. Peak inspiratory flow in patients with inadequate inhalation was significantly lower than that in patients with adequate inhalation, for all numbers of inhalation repeats analyzed. Receiver operating characteristic analyses indicated peak inspiratory flow cut-off values of 140, 120, 100, and 100 L/min at 1-4 successive inhalations, respectively.

**Conclusions:**

The present findings suggest that a proportion of patients with low peak inspiratory flow were unable to inhale the active ingredient adequately when laninamivir dry powder inhaler was administered as two successive inhalations, as recommended in the instruction manual. Three or four repeated inhalations of laninamivir dry powder inhaler should be administered to pediatric patients with low peak inspiratory flow.

**Electronic supplementary material:**

The online version of this article (10.1186/s40780-017-0094-7) contains supplementary material, which is available to authorized users.

## Background

Inavir® (Daiichi Sankyo Co., Ltd., Tokyo, Japan) is a disposable dry powder inhaler (DPI) formulation of laninamivir octanoate hydrate (laninamivir), a long-acting neuraminidase inhibitor, featuring two plastic medicine compartments (TwinCaps®, Hovione, Loures, Portugal) [1]. Laninamivir DPI is widely used for treating influenza virus A and B infections in Japan because of the convenient single-use treatment course [2]. However, if laninamivir DPIs are not operated correctly, a sufficient therapeutic effect cannot be obtained [3]. Therefore, pharmacists are required to instruct patients about the proper inhalation technique and provide information on the appropriate use of the inhalers [4].

The pharmaceutical company manufacturing laninamivir DPI suggests that pharmacists confirm patient inhalation ability and proper inhalation technique using a special whistle provided by the company when laninamivir DPI is prescribed to pediatric patients. Nevertheless, pediatric patients with low peak inspiratory flow (PIF) and those aged 4-6 years have been reported to be unable to adequately inhale laninamivir [3, 5]. In addition, decreased effectiveness of laninamivir and prolonged disease duration have been observed in patients with >20% of residual drug after inhalation [3]. Furthermore, for zanamivir DPI (Relenza®, GlaxoSmithKline), another inhaled formulation of an anti-influenza drug, it was shown that pediatric patients (≥ 5 years old) found inhaling the entire amount in a single inhalation difficult, indicating that repeated inhalations are needed to achieve sufficient therapeutic effect [6]. Therefore, we hypothesized that a single inhalation of laninamivir DPI may not achieve sufficient inhalation of the active ingredient in pediatric patients with a predictably low PIF [7, 8]. In support of this hypothesis, two successive inhalations (puffs) of laninamivir DPI have been recommended in the instruction manual by the manufacturer to reduce residual amounts of the drug. However, the appropriate number of inhalation repeats and factors influencing the residual amount of laninamivir DPI ingredients in pediatric patients have not been reported. In addition, clinical trials of Inavir® performed by the pharmaceutical company were not conducted using the TwinCaps® device [9] and it is unclear whether pediatric patients can properly use the inhaler. In the present study, we prospectively investigated the appropriate number of inhalation repeats and factors influencing the residual amount of laninamivir DPI ingredients in pediatric patients with influenza.

## Methods

### Study design

This prospective study enrolled 65 patients who received laninamivir DPI (Inavir®) between January and March 2016 at Tsu emergency medical center/pediatric clinic and dental clinic (Mie, Japan). Written informed consent was obtained from all study participants or guardians. One patient was unable to receive treatment via inhalation and was excluded. A pharmacist (with 7 years of work experience) instructed patients and/or their guardians in proper inhalation technique using leaflets describing the structure and inhalation procedure of laninamivir DPI (INAIP00104_0DH, Daiichi Sankyo Co., Ltd., Tokyo). Following confirmation of patient inhalation ability by the pharmacist (using a whistle provided by Daiichi Sankyo Co., Ltd., Tokyo, Japan), patients received laninamivir DPI treatment.

### Determination of the residual amounts of laninamivir dry powder

All enrolled patients performed four successive inhalations of laninamivir DPI following pharmacist instructions. One inhalation was defined as a single inhalation from two plastic dose compartments of the DPI. The residual amount of laninamivir dry powder was determined by measuring the device weight before and after each inhalation repeat using an electronic balance (LB-300 T, Takazono Co., Ltd., Tokyo, Japan). The residual amount of laninamivir dry powder was calculated by subtracting the device weight after inhalation from that before inhalation, as previously described [3]. If the residual amount was >20% compared to laninamivir dry powder content before use (0.1 g), inhalation was defined as inadequate.

### Data collection on eligible patients

Patient characteristics (age, sex, Rohrer index, and body temperature) were extracted from clinical records. Respiratory rate was estimated by observing the respiratory movements of the thorax in a sitting position. Percutaneous arterial oxygen saturation (SpO_2_) and pulse rate were determined using a portable pulse oximeter (BO-600, Japan Precision Instruments Inc., Shibukawa, Japan). History of asthma was obtained from patient interviews. PIF in a state of maximal inspiration before inhalation of laninamivir DPI was measured using an In-Check® oral inspiratory flow meter (accuracy: ± 10%, Clement Clarke International Ltd., Essex, UK) without any adapters. A paper disposable mouthpiece was attached to the In-Check® device for each patient.

### Statistical analyses

Statistical comparisons were performed using the Wilcoxon test with Bonferroni correction, Mann-Whitney U-test, and Fisher’s exact test for continuous and categorical variables, respectively. Cut-off values for continuous variables were determined by receiver operating characteristic (ROC) curve method. Statistical analyses were performed with JMP® version 12.0.1 (SAS Institute Inc., Cary, NC, USA). Significance was established at *P* < 0.05.

## Results

### Patient characteristics

Characteristics of the 64 enrolled participants are summarized in Table 1. The median age was 9 years [range: 5–15 years]. Thirty-seven patients (58%) were male. The median PIF value was 110 [range: 40–240 L/min].

**Table 1:** Patient characteristics

Characteristics	
Number of patients	64
Age (years)	9 [5 – 15]
Male	37 (58)
Rohrer index (kg/cm^3^)	126 [83 – 246]
Body temperature (°C)	38.4 [35.8 – 40.4]
History of asthma	11 (17)
Pulse rate (beats/min)	115 [73 – 150]
Respiratory rate (breaths/min)	25 [14 – 50]
SpO_2_ (%)	96 [93 – 99]
PIF (L/min)	110 [40 – 240]

Values are presented as median [range] or number (%)

SpO_2_: peripheral arterial oxygen saturation, *PIF* peak inspiratory flow

### Residual amounts of laninamivir dry powder after repeated inhalations

**Fig. 1:**
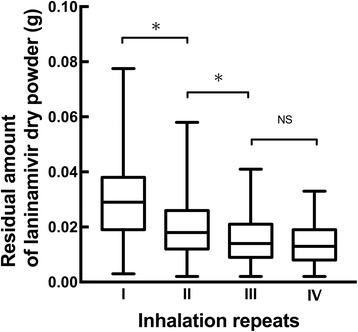
Residual amount of laninamivir dry powder after repeated inhalation of laninamivir DPI. Box plots represent the median with range. Statistical analyses were performed using the Wilcoxon test with Bonferroni correction. *: *P* < 0.05, NS: not significant

Figure 1 shows the residual amounts of laninamivir dry powder after each inhalation repeat. The median residual amounts [range] corresponding to one to four inhalation repeats were 0.029 [0.003 – 0.078 g], 0.019 [0.002 – 0.058 g], 0.014 [0.002 – 0.041 g], and 0.013 [0.002–0.033 g], respectively. Significant differences in residual drug amount were observed between inhalation repeats, except when three and four successive inhalations were compared.

### Inadequate inhalation rate after repeated inhalations

**Fig. 2:**
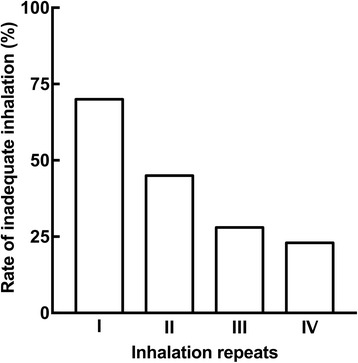
Rate of inadequate inhalation after repeated inhalations of laninamivir DPI

Figure 2 shows the rate of inadequate inhalation after each inhalation repeat. The inadequate inhalation rate was 70% after a single inhalation, and decreased with increased number of inhalation repeats (45, 28, and 23%, after two, three, and four inhalations, respectively).

### Comparisons of patient characteristics between adequate and inadequate inhalation groups after each inhalation repeat

Comparisons of patient characteristics between the adequate and inadequate inhalation groups after repeated inhalations are summarized in Table 2. PIF values in patients with inadequate inhalation were significantly lower than those in patients with adequate inhalation for each analyzed number of inhalation repeats. Moreover, the age of patients with inadequate inhalation was significantly lower than that of patients with adequate inhalation after two, three, and four repeated inhalations. Significantly fewer male patients displayed inadequate inhalation after one, two, or three successive inhalations. Furthermore, respiratory rates in patients who were unable to inhale adequately after a single inhalation were significantly higher than those in patients with adequate inhalation.

**Table 2:** Comparisons of patient characteristics between adequate and inadequate inhalation groups after repeated inhalations

Inhalation repeats	I		II		III		IV	
Groups by inhalation	Adequate	Inadequate	Adequate	Inadequate	Adequate	Inadequate	Adequate	Inadequate
	(*n* = 19)	(*n* = 45)	(*n* = 35)	(*n* = 29)	(*n* = 46)	(*n* = 18)	(*n* = 49)	(*n* = 15)
Age (years)	10 [5-14]	9 [5-15]	10 [5-15]	9 [5-14]*	10 [5-15]	7 [5-13]*	10 [5-15]	7 [5-13]*
Male	15 (79)	22 (49)*	26 (74)	11 (38)*	31 (67)	6 (33)*	31 (63)	6 (40)
Rohrer index (kg/cm^3^)	134 [83-188]	125 [98-246]	128 [83-246]	126 [98-190]	126 [83-246]	127 [100-190]	126 [83-246]	127 [100-190]
Body temperature (°C)	38.4 [35.9-40.4]	38.4 [35.8-40.3]	38.4 [35.9-40.4]	38.5 [35.8-40.1]	38.4 [35.9-40.4]	38.5 [35.8-40.0]	38.4 [35.9-40.4]	38.5 [35.8-40.0]
History of asthma	2 (11)	9 (20)	7 (20)	4 (14)	10 (22)	1 (6)	10 (20)	10 (67)
Pulse rate (beats/min)	116 [88-143]	115 [73-150]	114 [73-143]	117 [77-150]	114 [73-143]	119 [77-150]	114 [73-143]	119 [77-150]
Respiratory rate (breaths/min)	19 [14-35]	25 [14-50]*	22 [14-50]	25 [14-37]	23 [14-50]	26 [18-37]	23 [14-50]	26 [18-37]
SpO_2_ (%)	96 [94-98]	97 [93-99]	96 [94-99]	97 [93-99]	96 [93-99]	97 [94-99]	96 [93-99]	97 [94-99]
PIF (L/min)	150 [80-240]	110 [40-230]*	140 [80-240]	100 [40-200]*	130 [80-240]	85 [40-150]*	130 [80-240]	85 [40-150]*

Values are presented as median [range] or number (%). Statistical analyses were performed using Fisher’s exact test or Mann-Whitney U-test. *: *P* < 0.05. SpO_2_: peripheral arterial oxygen saturation, *PIF* peak inspiratory flow

### ROC analyses for PIF cut-off values after repeated inhalations

As significant differences in PIF values were observed for all numbers of inhalations analyzed, we calculated PIF cut-off values using ROC analyses. PIF cut-off values, area under the ROC curve (AUC), sensitivity, and specificity estimated by ROC analyses are summarized in Table 3. ROC analyses indicated PIF cut-off values of 140, 120, 100, and 100 L/min for one, two, three, and four inhalation repeats, respectively.

**Table 3:** ROC analyses for PIF cut-off values after repeated inhalations

Inhalation repeats	PIF cut-off value (L/min)	Sensitivity (%)	Specificity (%)	AUC	Positive predictive value (%)	Negative predictive value (%)
I	140	84	53	0.69	81	59
II	120	86	69	0.83	69	86
III	100	89	76	0.89	59	95
IV	100	87	71	0.86	48	95

*AUC* area under the ROC curve

## Discussion

The adequate number of inhalation repeats and factors influencing the residual amount of laninamivir dry powder when using laninamivir DPI have not been previously reported. Our results show that pediatric patients were often unable to adequately inhale the active ingredient with two successive inhalations of laninamivir DPI. In addition, this is the first study to demonstrate that repeated inhalation of laninamivir DPI (more than three times) could improve the rate of inhalation in pediatric patients with PIF > 100 L/min.

As shown in Fig. 2, the inadequate inhalation rate after two successive inhalations suggested by the manufacturer was 45%, revealing that approximately half of pediatric patients were unable to attain adequate inhalation. Furthermore, the inhalation rate improved with increasing inhalation repeats. Tabata et al. [6] reported that patients 5-16 years old could not inhale the entire dose amount of zanamivir (Relenza®), another inhaled anti-influenza agent. Moreover, Katsumi et al. [5] showed that some patients aged 4-6 years were not able to inhale the full amount of laninamivir with a single inhalation. These reports indicate that it may be difficult to achieve adequate inhalation of laninamivir in pediatric patients with a single inhalation.

To explore factors influencing the residual amount of laninamivir dry powder, patient characteristics were compared between adequate and inadequate inhalation groups for all numbers of inhalation repeats (Table 2). Patients with inadequate inhalation were significantly younger and significantly fewer were male, compared to patients with adequate inhalation, for almost all inhalation repeats analyzed (Table 2). Furthermore, the respiratory rate in patients with inadequate inhalation was higher than that in patients with adequate inhalation (Table 2). PIF was previously reported to be positively correlated with age in pediatric patients [8]. Moreover, it is considered that respiratory rate affects PIF value. Taylor et al. [10] reported sex as an independent factor contributing to PIF. In the present study, we confirmed a positive correlation between PIF and age (*r* = 0.514, *P* < 0.001, Additional file 1: Figure S1) as well as a negative correlation between PIF and respiratory rate (*r* = −0.403, *P* = 0.001, Additional file 2: Figure S2). Moreover, PIF values in female patients were significantly lower than those in male patients (*P* = 0.007, Additional file 3: Figure S3). Most importantly, PIF values in patients with inadequate inhalation were significantly lower than those in patients with adequate inhalation, regardless of the number of inhalation repeats. These observations suggest that PIF is the most suitable indicator for evaluating residual drug amounts. Additionally, as female or younger patients, and/or patients with a high respiratory rate, are unlikely to adequately inhale laninamivir DPI, inhalation should be monitored in these patients when PIF is not determined.

To determine the appropriate number of inhalation repeats and select values of PIF useful as a clinical indicator, PIF cut-off values were calculated using ROC analyses (Table 3). These results suggest that two successive inhalations are sufficient for patients with PIF > 120 L/min, whereas at least three inhalation repeats are required for patients with lower PIF (100-120 L/min).

This study indicated that inhalation of laninamivir dry powder appears insufficient in patients with PIF < 100 L/min (Table 3), in agreement with a previous study, which demonstrated that patients with PIF < 90 L/min could not adequately inhale laninamivir dry powder [3]. Considering the accuracy (± 10%) of the In-Check® inspiratory flow meter used, the differences in these PIF values are within expectations. As judging whether administration of treatment by inhalation is suitable using only the whistle designed for confirmation of inhalation ability is considered difficult, additional assessments based on PIF may allow more reliable evaluation of inhalation applicability.

The limitations of our present study include the small number of cases, not examining repeated inhalations according to patient’s individual understanding of the inhalation technique, and not evaluating the therapeutic efficacy of laninamivir.

## Conclusions

Our present findings suggest that some pediatric patients are unable to adequately inhale the active ingredient after two successive inhalations of laninamivir DPI. Pediatric patients with low PIF may require more than three repeated inhalations of laninamivir DPI. These results provide useful information for the successful use of laninamivir DPI in pediatric patients with influenza.

## Additional files

Additional file 1: Figure S1. Correlation between PIF and age in pediatric patients receiving laninamivir dry powder inhaler (*n* = 64). Statistical analysis was performed using Spearman correlation coefficient. Each point represents the patients. PIF, peak inspiratory flow. (PDF 716 kb)

Additional file 2: Figure S2. Correlation between PIF and respiratory rate in pediatric patients receiving laninamivir dry powder inhaler (*n* = 64). Statistical analysis was performed using Spearman correlation coefficient. Each point represents the patients. (PDF 562 kb)

Additional file 3: Figure S3. Comparisons of PIF between male (*n* = 37) and female (*n* = 27) pediatric patients receiving laninamivir dry powder inhaler. Statistical analysis was performed using Mann-Whitney *U* test. Each point represents the patients and median are indicated by horizontal lines. PIF, peak inspiratory flow. (PDF 738 kb)

## Abbreviations

AUC: Area under the ROC curve
DPI: Dry powder inhaler
PIF: Peak inspiratory flow
ROC: Receiver operating characteristic
SpO_2_: Percutaneous arterial oxygen saturation
