# Supplementary material for: Impact of the number of repeated inhalations and patient characteristics on the residual amount of inhaled laninamivir octanoate hydrate dry powder in pediatric patients with influenza
Source: J Pharm Health Care Sci. 2017 Nov 8;3:26. doi: 10.1186/s40780-017-0094-7 (PMC5678805; doi:10.1186/s40780-017-0094-7)
Supplement: Supplementary file 1 — Correlation between PIF and age in pediatric patients receiving laninamivir dry powder inhaler (n = 64). Statistical analysis was performed using Spearman correlation coefficient. Each point represents the patients. PIF, peak inspiratory flow. (PDF 716 kb) [file 40780_2017_94_MOESM1_ESM.pdf]

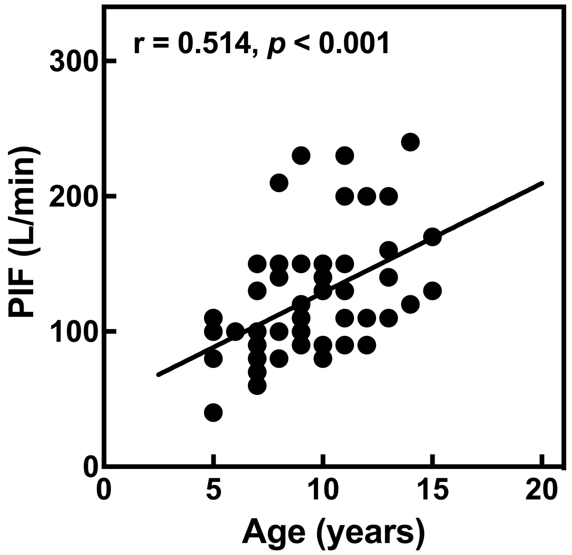


**Additional file 1: Figure S1 Correlation between PIF and age in pediatric patients receiving laninamivir dry powder inhaler (n = 64).** Statistical analysis was performed using Spearman correlation coefficient. Each point represents the patients. PIF, peak inspiratory flow
