# Supplementary material for: Impact of the number of repeated inhalations and patient characteristics on the residual amount of inhaled laninamivir octanoate hydrate dry powder in pediatric patients with influenza
Source: J Pharm Health Care Sci. 2017 Nov 8;3:26. doi: 10.1186/s40780-017-0094-7 (PMC5678805; doi:10.1186/s40780-017-0094-7)
Supplement: Supplementary file 2 — Correlation between PIF and respiratory rate in pediatric patients receiving laninamivir dry powder inhaler (n = 64). Statistical analysis was performed using Spearman correlation coefficient. Each point represents the patients. (PDF 562 kb) [file 40780_2017_94_MOESM2_ESM.pdf]

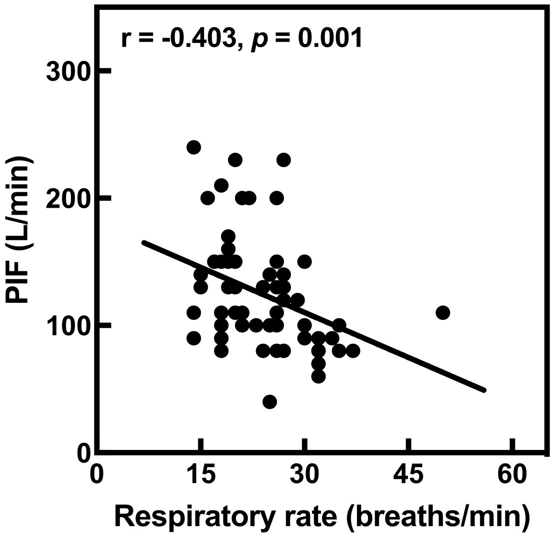

Additional file 2: Figure S2 Correlation between PIF and respiratory rate in pediatric patients receiving laninamivir dry powder inhaler (n = 64). Statistical analysis was performed using Spearman correlation coefficient. Each point represents the patients.
