# Supplementary material for: Impact of the number of repeated inhalations and patient characteristics on the residual amount of inhaled laninamivir octanoate hydrate dry powder in pediatric patients with influenza
Source: J Pharm Health Care Sci. 2017 Nov 8;3:26. doi: 10.1186/s40780-017-0094-7 (PMC5678805; doi:10.1186/s40780-017-0094-7)
Supplement: Supplementary file 3 — Comparisons of PIF between male (n = 37) and female (n = 27) pediatric patients receiving laninamivir dry powder inhaler. Statistical analysis was performed using Mann-Whitney U test. Each point represents the patients and median are indicated by horizontal lines. PIF, peak inspiratory flow. (PDF 738 kb) [file 40780_2017_94_MOESM3_ESM.pdf]

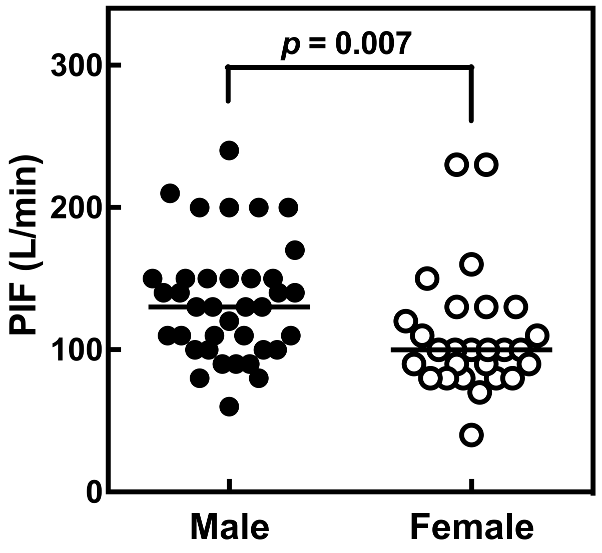

Additional file 3: Figure S3 Comparisons of PIF between male (n = 37) and female (n = 27) pediatric patients receiving laninamivir dry powder inhaler.

Statistical analysis was performed using Mann-Whitney *U* test.

Each point represents the patients and median are indicated by horizontal lines.

PIF, peak inspiratory flow
